# HSP70: From Signaling Mechanisms to Therapeutics

**DOI:** 10.3390/biom13071141

**Published:** 2023-07-17

**Authors:** Kenia Pedrosa Nunes, Amanda Almeida de Oliveira

**Affiliations:** Department of Biomedical Engineering and Sciences, Florida Institute of Technology, Melbourne, FL 32901, USA

Heat-shock proteins (HSPs) are primary stress responders that are vital to maintaining homeostasis [1]. HSP70 (also known as HSPA) is a family of highly preserved HSPs that exerts many biological functions in health and disease [2]. In humans, the HSP70 family encompasses 13 homologous genes, which are expressed in a tissue-dependent manner and occupy various intracellular (iHSP70) compartments, such as the cytosol, mitochondria, lysosomes, and nucleus [3]. Adding to the complexity of this field, HSP70 can be detected in the extracellular (eHSP70) space, acting as an endogenous stress sensor [4,5,6,7]. Therefore, current research in this field (HSP70) spans from the search for novel therapeutics to the exploitation of new biomarkers. In this sense, this Special Issue includes a set of original research/review articles that summarize recent discoveries relating HSP70 to various conditions, including hypertension [7], vascular dysfunction associated with aging [8], cancer [9,10,11], atherosclerotic cardiovascular disease [12], and COVID-19 [13]. 

As discussed by Rodriguez-Iturbe and colleagues, in hypertension, HSP70 exerts both chaperone and cytokine functions and may induce, depending on the context, tolerogenic anti-inflammatory reactivity or immunogenic and autoimmune reactivity [7]. Low-grade sterile inflammation is a hallmark of hypertension, caused by the activation of innate and adaptative immune mechanisms [6,7]. One of the critical details highlighted is that the HSP70-mediated stress within an individual, whether pro-inflammatory or anti-inflammatory, is crucial to determining the progression of or protection from hypertension [7]. Additionally, HSP70 has emerged as a critical participant in the vasculature [14] due to its interaction with calcium-handling mechanisms [15] in a sex-dependent manner [16]; it might also utilize alternative routes in order to influence the pathophysiology of vascular diseases, such as hypertension. In this context, de Oliveira and collaborators demonstrated that age-related alterations in HSP70 are associated with a reduction in vascular responses to adrenergic stimulation, and consequently, to the impairment of arterial function [8]; this may be a factor influencing vascular aging, and in some cases, present a link between aging and comorbidities, including hypertension.

There is a dichotomy between the compartmentalization of HSP70 and its biological actions, with iHSP70 playing protective roles and eHSP70 promoting cell/tissue damage [2,4]. To date, the literature supports that, in cardiovascular diseases and with regard to cardiovascular risk factors, diminished iHSP70 and increased eHSP70 have detrimental effects. These findings have prompted the search for ways to shift the balance towards higher iHSP70 levels. As discussed by Nagai and Kaji, levels of HSP70 may be enhanced in skeletal muscle via exercise or thermal stimulation [12]. In fact, the authors emphasize that thermal stimulation protects against insulin resistance, suppresses skeletal muscle atrophy, and has anti-apoptotic and anti-inflammatory actions, which are factors associated with atherosclerotic cardiovascular diseases. While brief, this review elucidates the issue of the thermal modulation of HSP70 as a tool with which to prevent cardiovascular complications. Expanding the contributions of HSP70, and in light of the COVID-19 pandemic, Russo and colleagues [13] reported that severe COVID-19 patients present elevated eHSP70 levels and an impaired heat-shock response capacity, which may have implications for disease severity, plus long-term effects regarding disease susceptibility; this highlights yet another exciting avenue for research.

HSP70 is also associated with a vast number of cancer types, and often, its overexpression is linked to a poor prognosis [17]. In this context, HSP70 acts as a chaperone molecule while also exerting a regulatory function in essential signaling pathways within the tumor microenvironment. Such roles justify the many ongoing clinical trials considering the possibility of HSP70-based monotherapy via HSP70 inhibitors or in combination with other drugs [10]. An elegant study by Safi and collaborators [9] propounded that the level of serum HSP72 is a possible biomarker for advanced lung cancer, as it might potentially enable lung cancer and metastatic disease to be distinguished between. Although this study remains to be validated in a larger cohort of patients, to obtain a foretelling signature that can be employed to screen for lung cancer, or any other cancer type, would be a milestone in this field. Despite extensive data characterizing HSP70 as an ally in cancer management being available and the positive effects when this protein is blocked being exhibited, to date, inhibitors for HPS70 are not available in clinical practice [11]. As discussed by Mouawad and colleagues, in the context of onco-hematological diseases (and potentially other cancer types), a major challenge is the off-target effects of HSP70 pharmacological modulators, as HSP70 is a pervasive protein exerting multiple functions across all human systems. Thus, inhibiting this protein without compromising healthy cells is paramount; however, currently, it is extremely challenging.

In essence, the HSP70 research field is vast, and fulfils the diverse set of articles included in this Special Issue; this reinforces the dynamism of HSP70, while providing an insight into the challenges involved in transferring HSP70 from the bench to the bedside. Nevertheless, we firmly believe that the above-mentioned articles enhance our understanding of HSP70-mediated processes in human diseases; this is accomplished by providing insights into knowledge gaps, and hopefully, devising directions for future research in the field.
